# Experiences of commissioning mental health services for children and young people in England: qualitative study of commissioners’ perspectives

**DOI:** 10.1192/j.eurpsy.2024.405

**Published:** 2024-08-27

**Authors:** S. P. Trethewey, K. A. Allen, F. Matthews, A. Price, T. Newlove-Delgado

**Affiliations:** Children and Young People’s Mental Health (ChYMe) research collaboration, University of Exeter, Exeter, United Kingdom

## Abstract

**Introduction:**

Commissioners play a central role in coordinating and planning CAMHS. However, there is little research on their experiences and approaches to understanding the needs of their populations. An improved understanding is likely to benefit the translation of research into practice, by ensuring research outputs meet the needs of key stakeholders and in optimising the sharing and use of data to improve services.

**Objectives:**

To better understand commissioners’ experiences of commissioning child and adolescent mental health services (CAMHS) and the challenges they face.

**Methods:**

Between May to June 2023, we conducted twelve individual, semi-structured interviews with Integrated Care Board commissioners of CAMHS across England. We analysed data using framework analysis; a qualitative analysis method which involves systematically charting and organising data using a framework to generate themes.

**Results:**

We generated five core themes from the data: 1) ‘Reflections on role’ – how commissioners’ roles are informed by their background and ‘positioning’ within the system in which they work, 2) ‘Priorities and Tensions’ – the wider context in which commissioners work and how this may present challenges, 3) ‘Insights and evidence’– how commissioners develop an understanding of child mental health need and the different roles of quantitative and qualitative data, 4) ‘Children’s mental health in the limelight’ – commissioners’ perceptions of changes in child mental health in their populations, 5) ‘Responding to need’ – how commissioners are addressing the needs of their populations and the challenges they perceive.

**Conclusions:**

CAMHS commissioners are negotiating a complex and changing political, social and economic environment with differing priorities and pressures. Commissioners draw heavily on insights from providers and their role is shifting towards managing relationships and bringing the system together. A key challenge is balancing investment in prevention/early intervention versus specialist services needed by children with more severe and complex problems.

**Disclosure of Interest:**

None Declared

